# Interaction of 5′-Guanosine Monophosphate with Organotin(IV) Moieties: Synthesis, Structural Characterization, and Anti-Inflammatory Activity

**DOI:** 10.5402/2012/873035

**Published:** 2012-09-30

**Authors:** Mala Nath, Hitendra Singh, George Eng, Xueqing Song

**Affiliations:** ^1^Department of Chemistry, Indian Institute of Technology Roorkee, Uttrakhand, Roorkee 247667, India; ^2^Department of Chemistry and Physics, University of the District of Columbia, Washington, DC 20008, USA

## Abstract

Reaction(s) of 5′-guanosine monophosphate (5′GMP) with di- and triorganotin(IV) chloride(s) led to formation of organotin(IV) derivatives of general formulae, [R_2_Sn(5′-GMP)*·*H_2_O]_*n*_ and [(R′_3_Sn)_2_(5′-GMP)*·*H_2_O]_*n*_, where R = Me, *n*-Bu, and Ph; R′ = Me, *i*-Pr, *n*-Bu, and Ph; (5′-GMP)^2−^ = 5′-guanosine monophosphate. An attempt has been made to prove the structures of the resulting derivatives on the basis of FT-IR, multinuclear ^1^H, ^13^C, and ^119^Sn NMR and ^119^Sn Mössbauer spectroscopic studies. These investigations suggest that both di- and triorganotin(IV)-5′-guanosine monophosphates are polymeric in which (5′-GMP)^2−^ is bonded through phosphate group resulting in a distorted trigonal bipyramidal geometry around tin. The ribose conformation in all of the derivatives is *C*3′-*endo*, except diphenyltin(IV) and tri-*i*-propyltin(IV) derivatives where it is *C*2′-*endo*. All of the studied derivatives exhibited mild-to-moderate anti-inflammatory activity (~15.64–20.63% inhibition) at 40 mg kg^−1^ dose and LD50 values > 400 mg kg^−1^ in albino rats.

## 1. Introduction

The field of cancer chemotherapy has been developed enormously during the past fifty years. Prior to 1969, however, the arsenal of chemotherapeutic agents was devoid of compounds which are inorganic in nature because of generally accepted belief that most metals and their compounds were potentially carcinogenic [1]. In 1969, Rosenberg and his coworkers made the serendipitous discovery [2] that certain Pt compounds were potent antitumor agents against Sarcoma 180 tumors and L1210 leukemia in mice and must be considered to be an outstanding development in the field of metal compounds in medicine [3]. *Cis*-platin is the first drug from inorganic chemistry to have come under routine clinical use in medical oncology [3]; in 1986, it was the largest selling anticancer drug worldwide. Its success placed the co-ordination chemists on the front line in the fight against cancer and stimulated the search for other metal-containing compounds with potential anticancer activity. In last 20 years about more than 12000 complexes of 55 metals have been tested [4], many of them are now entering for clinical trials, and some may ultimately rival *cis*-platin [5–7]. Although the majority of these successes involved complexes containing transition metal ions such as Cr, Co, Cu, Pd, Rh, Ru, and Au [5–8], but some main group metals (i.e., Al, Ga, In, ad Tl; Ge, Sn, and Pb; Bi and Po) compounds [1], especially organotins, have also been discovered which show promise as future members of man's anticancer arsenal [9–13]. Further, several organotin(IV) derivatives have been reported to exhibit good anti-inflammatory activity [14–22].

The mechanism of mode of action of* cis*-platin is due to the formation of an intrastrand crosslink with DNA, involving the N7 of two guanine residues [23, 24]. The mode of action of organotin compounds is not very well documented. In order to obtain a better insight about the interaction of organotins with DNA inside the biological systems, their studies with basic constituent units of DNA are indispensable. In view of this, some studies on organotin-nucleotides both in solid-state and in solution have been carried out [25–30]. Stannylated ribonucleotides in the presence of iodine as activating agent have been used in chemical synthesis of m^7^G^5′^ppNu (Nu = A, G, C, and U) [31]. In continuation to our recent studies on the interaction of organotin(IV) moieties with guanine [21] and guanosine [22], in this paper, we wish to report the results of the interaction of 5′-guanosine monophosphate with tri- and diorganotin(IV) moieties.

## 2. Experimental

Solvents were dried and distilled before use. Dimethyltin(IV) dichloride di-*n*-butyltin(IV) dichloride, diphenyltin(IV) dichloride, trimethyltin(IV) chloride, tri-*i*-propyltin(IV) chloride, tri-*n*-butyltin(IV) chloride, triphenyltin(IV) chloride (E. Merck), di-*n*-octyltin(IV) oxide (Aldrich), and disodium salt of 5′-guanosine monophosphate (Na_2_(5′-GMP) (Sigma) were used as received. The elemental analysis, namely, melting points, carbon, hydrogen, nitrogen, and tin of the synthesized compounds was determined on the same instruments as reported earlier [21, 22]. Infrared and far-infrared spectra were recorded on a Perkin-Elmer 1600 series FT IR spectrophotometer in the range 4000–400 cm^−1^ from KBr discs and 600–200 cm^−1^ from CsI discs. ^1^H and ^13^C spectra were recorded on a Bruker DRX 300 (300 MHz FT NMR) spectrometer at the Central Drug Research Institute, Lucknow, India, using CD_3_OD as solvent and TMS as the internal standard. ^119^Sn NMR spectra were recorded on a Bruker DRX 500 (500 MHz FT NMR) spectrometer at the Institute Instrumentation Centre, IIT, Roorkee, India, using DMSO-d_6_/CD_3_OD as solvent and TMS as the internal standard [21, 22]. ^119^Sn Mössbauer spectra were recorded on Mössbauer spectrometer model MS-900 according to the procedure reported previously at the Department of Chemistry and Physics, University of The District of Columbia, Washington, DC, USA [21, 22]. Toxicity (LD_50_: average lethal dose at 50% survival) and anti-inflammatory activity of the studied derivatives were determined according to the procedures reported earlier [21, 22]. 

### 2.1. General Method for Synthesis of Dimethyltin/di-*n*-butyltin/diphenyltin(IV) Derivatives of (5′-GMP)^2−^

Na_2_(5′-GMP) (0.814 g, 2.0 mmol) was dissolved in the minimum amount (20 mL) of aqueous methanol (1 : 1 or 50%). The resulting solution was refluxed for half an hour with constant stirring. To this it was added an aqueous methanol (20 mL, 1 : 1) solution of dimethyltin(IV) dichloride (0.440 g, 2.0 mmol)/di-*n*-butyltin(IV) dichloride (0.608 g, 2.0 mmol)/diphenyltin(IV) dichloride (0.688 g, 2.0 mmol) at room temperature (30 ± 2°C). The resulting solution was further refluxed with constant stirring for another ~20 h for di-*n*-butyl/diphenyltin(IV) derivatives, whereas only stirring was carried out at room temperature for dimethyltin(IV) derivative. The solid product thus obtained was washed with water and then with methanol-hexane or methanol-petroleum ether (b.p. 40–60°C) mixture (1 : 3 v/v) and dried under vacuum. 

### 2.2. Physical Characteristic and Infrared Spectral Data for Dimethyltin/Di-*n*-butyltin/Diphenyltin(IV) Derivatives of (5′-GMP)^2−^

[Me_2_Sn(5′-GMP)·H_2_O]_*n*_ (**1**): white solid; yield, 65%; m.p. 275−278 (dec.)°C. Elemental Anal. Calc. for [C_12_H_20_N_5_O_9_PSn]_*n*_: C 27.30, H 3.82, N 13.26, Sn 22.48%. Found: C 27.03, H 3.57, N 13.01, Sn 22.13%. IR: *ν*(NH_2_)+*ν*(OH), 3426 s, 3309 s, 3233 s; *ν*(C=O), 1717 vs; *δ*(NH_2_), 1635 vs; *ν*(C=N) + *ν*(C=C), 1600 s, 1565 s; *ν*(CO) in ribose, 1113 vs; *ν*_as_ (PO_3_)^2-^/*ν*_s_ (PO_3_)^2-^, 1080 s, 1009 s, 925 w; ribose pucker, 791 s; *ν*_as_ (Sn−C)/*ν*_s_ (Sn−C), 605 w, 565 sh, 530 m; *ν*(Sn−O)/*ν*(Sn−O−Sn), 452 m. 

[*n*-Bu_2_Sn(5′-GMP)·H_2_O]_*n*_ (**2**): white solid; yield 72%; m.p. 250−255 (dec.)°C, reported m.p. 220 (dec)°C [29]. Elemental Anal. Calc. for [C_18_H_32_N_5_O_9_PSn]_*n*_: C 35.32, H 5.27, N 11.44, Sn 19.39%. Found: C 35.11, H 5.09, N 11.21, Sn 19.07%. IR: *ν*(NH_2_)+*ν*(OH), 3400 brs, 3230 sh, 3137 s; *ν*(CO), 1695 vs; *δ*(NH_2_), 1650 sh; *ν*(C=N) + *ν*(C=C), 1612 m, 1585 sh, 1533 m; *ν*(CO) in ribose, 1106 m; *ν*_as_ (PO_3_)^2-^/*ν*_s_ (PO_3_)^2-^, 1075 m, 1020 w, 977 m; ribose pucker, 803 m; *ν*_as_ (Sn−C)/*ν*_s_ (Sn−C), 577 w, 512 w; *ν*(Sn−O)/*ν*(Sn−O−Sn), 512 w. 

[Ph_2_Sn(5′-GMP)·H_2_O]_*n*_ (**3**): cream solid; yield, 73%; m.p. 150−155 (dec.)°C. Elemental Anal. Calc. for [C_22_H_24_N_5_O_9_PSn]_*n*_: C 40.52, H 3.71, N 10.74, Sn 18.20%. Found: C 40.29, H 3.46, N 10.57, Sn 17.91%. IR: *ν*(NH_2_)+*ν*(OH), 3413 sbr, 3362 s, 3222 sh; *ν*(CO), 1689 vs; *δ*(NH_2_), 1635 vs; *ν*(C=N) + *ν*(C=C), 1598 sh, 1535 vw; *ν*(CO) in ribose, 1125 vs; *ν*_as_ (PO_3_)^2-^/*ν*_s_ (PO_3_)^2-^, 1023 s, 905 w; ribose pucker, 860 w; *ν*_as_ (Sn−C)/*ν*_s_ (Sn−C), 280 m, 222 w; *ν*(Sn−O)/*ν*(Sn−O−Sn), 509 m. 

### 2.3. General Method for Synthesis of Triorganotin(IV) Derivatives of (5′-GMP)^2−^

The procedure for the syntheses of triorganotin(IV) derivatives of (5′-GMP)^2−^ was same as discussed in the previous paragraph using the stoichiometric ratio of Na_2_(5′-GMP) and triorganotin(IV) chloride equal to 2 : 1. 

### 2.4. Physical Characteristic and Infrared Spectral Data for Triorganotin(IV) Derivatives of (5′-GMP)^2−^

[(Me_3_Sn)_2_(5′-GMP)·H_2_O]_*n*_ (**4**): white solid; yield 79%; m.p. 265−268 (dec.)°C. Elemental Anal. Calc. for [C_16_H_32_N_5_O_9_PSn_2_]_*n*_: C 27.19, H 4.56, N 9.91, Sn 33.59%. Found: C 26.85, H 4.26, N 9.73, Sn 33.30%. IR: *ν*(NH_2_)+*ν*(OH), 3430 s, 3130 m; *ν*(CO), 1691 vs; *δ*(NH_2_), 1639 w; *ν*(C=N) + *ν*(C=C), 1600 sh, 1535 w; *ν*(CO) in ribose, 1150 w; *ν*_as_ (PO_3_)^2-^/*ν*_s_ (PO_3_)^2-^, 1065 s, 986 m; ribose pucker, 800 m; *ν*_as_ (Sn−C)/*ν*_s_ (Sn−C), 605 w, 513 w; *ν*(Sn−O)/*ν*(Sn−O−Sn), 475 sh. 

[(*i*-Pr_3_Sn)_2_(5′-GMP)·H_2_O]_*n*_ (**5**): white solid; yield 81%; m.p. 212−215 (dec.)°C. Elemental Anal. Calc. for [C_28_H_56_N_5_O_9_PSn_2_]_*n*_: C 38.43, H 6.45, N 8.00, Sn 27.13%. Found: C 38.17, H 6.18, N 7.71, Sn 26.89%. IR: *ν*(NH_2_)+*ν*(OH), 3439 sh, 3352 sbr, 3217 w, 3143 sh; *ν*(CO), 1687 vs; *δ*(NH_2_), 1630 vs; *ν*(C=N) + *ν*(C=C), 1598 sh, 1535 m; *ν*(CO) in ribose, 1115 sh, 1155 w; *ν*_as_ (PO_3_)^2-^/*ν*_s_ (PO_3_)^2-^, 1078 s, 996 m; ribose pucker, 809 w; *ν*_as_ (Sn−C)/*ν*_s_ (Sn−C), 610 w, 517 m; *ν*(Sn−O)/*ν*(Sn−O−Sn), 470 m. 

[(*n*-Bu_3_Sn)_2_(5′-GMP)·H_2_O]_*n*_ (**6**): white solid; yield 72%; m.p. 190−195 (dec.)°C, reported m.p. 195 (dec)°C [29]. Elemental Anal. Calc. for [C_34_H_68_N_5_O_9_PSn_2_]_*n*_: C 42.57, H 7.30, N 7.14, Sn 24.75%. Found C 42.21, H 7.06, N 6.76, Sn 24.48%. IR: *ν*(NH_2_)+*ν*(OH), 3430 sbr, 3345 sh, 3117 s; *ν*(CO), 1691 s; *δ*(NH_2_), 1650 sh; *ν*(C=N) + *ν*(C=C), 1609 m, 1580 sh, 1539 s; *ν*(CO) in ribose, 1148 s; *ν*_as_ (PO_3_)^2-^/*ν*_s_ (PO_3_)^2-^, 1074 vs, 996 s; ribose pucker, 822 m; *ν*_as_ (Sn−C)/*ν*_s_ (Sn−C), 609 m, 513 m; *ν*(Sn−O)/*ν*(Sn−O−Sn), 461 w. 

[(Ph_3_Sn)_2_(5′-GMP)·H_2_O]_*n*_ (**7**): cream solid; yield 71%; m.p. 235−240 (dec.)°C. Elemental Anal. Calc. for [C_46_H_44_N_5_O_9_PSn_2_]_*n*_: C 51.19, H 4.11, N 6.49, Sn 21.99%. Found: C 50.83, H 3.88, N 6.33, Sn 21.78%. IR: *ν*(NH_2_)+*ν*(OH), 3422 sbr, 3213 sh, 3130 s; *ν*(CO), 1691 vs; *δ*(NH_2_), 1635 m; *ν*(C=N) + *ν*(C=C), 1604 m, 1535 m; *ν*(CO) in ribose, 1143 m; *ν*_as_ (PO_3_)^2-^/*ν*_s_(PO_3_)^2-^, 1065 vs, 996 s; ribose pucker, 800 m; *ν*_as_ (Sn−C)/*ν*_s_ (Sn−C), 276 vsm, 227 m; *ν*(Sn−O)/*ν*(Sn−O−Sn), 509 m.

## 3. Results and Discussion

**Scheme 1:**
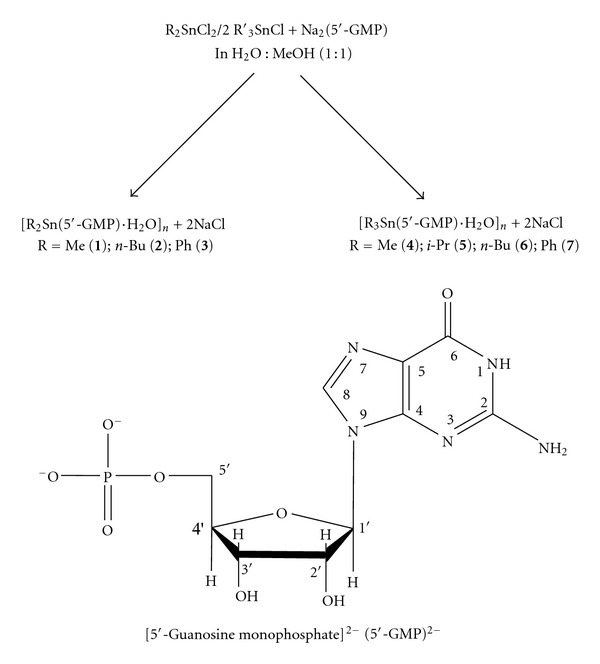
Reaction path ways for di- and triorganotin(IV) derivatives of (5′-GMP)^2−^.

Reactions of R_2_SnCl_2_ (R = Me, *n*-Bu, and Ph) or R_3_′SnCl (R′ = Me, *i*-Pr, *n*-Bu, and Ph) (aqueous methanol (50%) solution) with Na_2_(5′-GMP) in a 1 : 1 and 2 : 1 molar ratio, respectively, led to the formation of organotin(IV) derivatives **1**–**7** according to Scheme 1.

All of the synthesized compounds are obtained as white or cream solid in 65–81% yield and stable towards air and moisture. They are insoluble in common organic solvents but sparingly soluble in DMSO. They decomposed at high temperature instead of melting, which indicates their polymeric nature. The analytical data of all of the newly synthesized derivatives of (5′-GMP)^2−^ suggest that the resulting complexes are crystallized with 1 : 1 stoichiometry in case of diorganotin(IV) derivatives of (5′-GMP)^2−^, whereas 2 : 1 (Sn: 5′-GMP^2-^) stoichiometry is observed for triorganotin(IV) derivatives of (5′-GMP)^2−^. In the entire studied derivatives one molecule of water is also involved.

In the infrared spectra of di- and triorganotin(IV) derivatives of (5′-GMP)^2−^, three bands due to the *ν*(NH_2_) and *ν*(OH) are observed in the 3117–3362 cm^−1^ region as compared to a single broadband observed at 3314 cm^−1^ in Na_2_(5′-GMP). Further, NH_2_ deformation vibration undergoes some shifts (~±10 cm^−1^) in these organotin(IV) derivatives as compared to Na_2_(5′-GMP) (1638 cm^−1^). These shifts may be due to the different extent of hydrogen bonding in organotin(IV) derivatives in the solid-state. An additional band observed beyond 3400 cm^−1^ in these complexes indicates the presence of water molecule. The *ν*(C=O) stretching frequencies observed at 1690 cm^−1^ in Na_2_(5′-GMP) remains almost unchanged upon complexation. The *ν*(CO) of the hydroxyl group (–OH) of the ribofuranose residue in Na_2_(5′-GMP) appears at 1116 cm^−1^. All of the diorganotin(IV) derivatives of (5′-GMP)^2−^ exhibit *ν*(CO) frequencies in the region 1106–1125 cm^−1^, whereas all of the triorganotin(IV) derivatives are shown in the region 1143–1155 cm^−1^. These shifts may be attributed to a change in conformation in the ribose ring, and larger shifts in the triorganotin(IV) derivatives may be due to the possibility of bonding of second R_3_Sn(IV) group to the 3′-O, which is in agreement with reported value (1143 cm^−1^) [29] for (*n-*Bu_3_Sn)_2_(5′-GMP)·H_2_O. Ribose pucker marker bands have been reported in the 800–850 cm^−1^ region [29] with a band at ~800 cm^−1^ associated with the C3′-*endo* and at ~>820 cm^−1^ associated with the C2′-*endo*, the two most commonly found ribose puckers in nucleotides and nucleic acids. Ph_2_Sn(IV) and *iso*-Pr_3_Sn(IV) derivatives have ribose pucker band at 860 and 822 cm^−1^, respectively, whereas all other complexes show this band at 805 ± 5 cm^−1^, which indicate the C2′-*endo* conformation in the former and C3′-*endo* in the latter complexes.

The symmetric stretching vibration of the phosphate group (PO_3_)^2−^ of Na_2_(5′-GMP) gets shifted towards higher wave number except in [Ph_2_Sn(5′-GMP)·H_2_O]_*n*_ upon complexation, whereas the smaller shifts are also observed for the asymmetric stretching vibrations in all of the studied complexes, which indicate the bonding of the phosphate group with the organotin moiety. The appearance of new bands of medium intensity in the region 452–512 cm^−1^ in the studied complexes, which may be assigned to *ν*(Sn–O), further confirms the coordination of the (PO_3_)^2−^, group of (5′-GMP)^2−^ to tin through covalent bonding [29]. Therefore, coordination of (5′-GMP)^2−^ through NH_2_ and C=O groups of nucleobase is unlikely. The *ν*(Sn–C_2_) bands observed at around 594 ± 17 cm^−1^ and 521 ± 9 cm^−1^ can be identified as *ν*_as_ (Sn–C) and *ν*_s_(Sn–C), respectively, which is consistent with the *cis*-disposition of alkyl groups, whereas for the phenyl derivatives, the corresponding *ν*(Sn–C_2_) stretching bands are observed in the far-IR region of 222–280 cm^−1^ [21, 22].

The ^119^Sn Mössbauer spectral data of the studied compounds are presented in Table 1. The structures of R_2_Sn(IV) and R_3_Sn(IV) derivatives of (5′-GMP)^2−^ are considerably more complex than those of guanosine [22]. The ^119^Sn Mössbauer spectra of di- and trialkyltin(IV) derivatives of (5′-GMP)^2−^ exhibit a doublet centered (IS) at 1.14 ± 1 and 1.40 mm s^−1^, respectively, and quadrupole splitting in the range 3.24–3.55 mm s^−1^ and 3.30–3.35 mm s^−1^, respectively, while the IS and QS values for [Ph_2_Sn(5′- GMP)·H_2_O]_*n*_ are 0.61 mm s^−1^ and 1.83 mm s^−1^, respectively, and those of [(Ph_3_Sn)_2_(5′-GMP)·H_2_O]_*n*_ are 0.95 mm s^−1^ and 2.52 mm s^−1^, respectively. This suggests that the electric field gradient around the tin nucleus is generated by unequal electron densities in the tin-nucleotide bonds like tin-peptide [13, 17, 32] and is also due to the geometric distortions. The *ρ* (QS/IS) values (>2.0 in all of the R_2_Sn(IV)/R_3_Sn(IV) derivatives) suggest a coordination number of tin greater than four, and a significant line intensity asymmetry (the Goldanskii-Karyagin effect) (except [Me_2_Sn(5′-GMP)·H_2_O]_*n*_) suggests an intermolecularly associated lattice [13, 17, 32]. 

**Figure 1:**
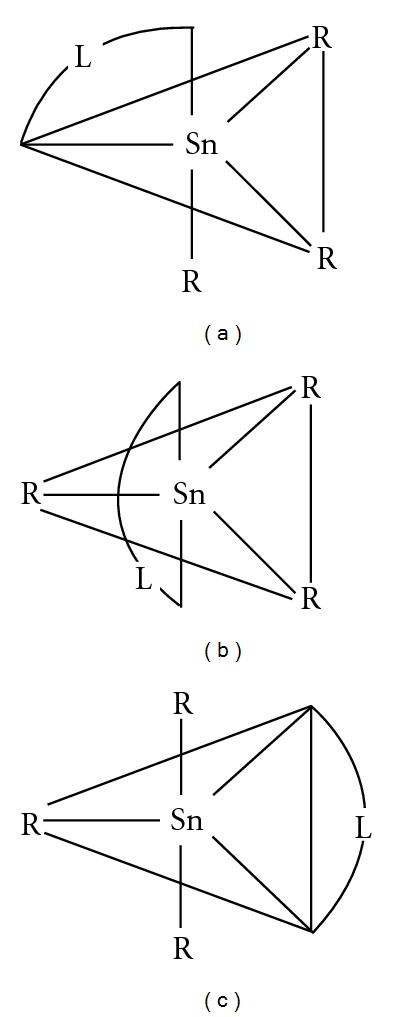
Possible isomers of R_3_SnL (where L = a bidentate ligand).

**Figure 2:**
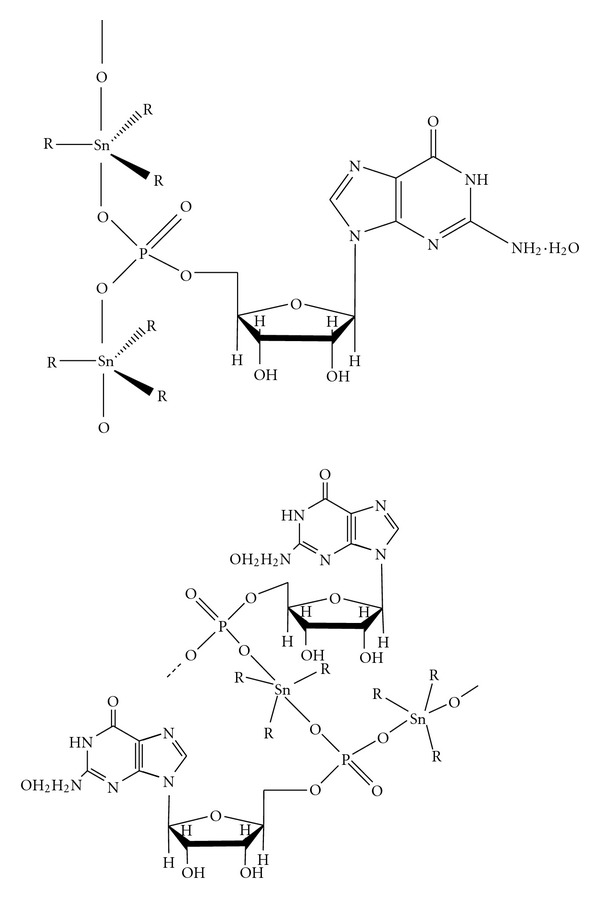
Proposed structures of triorganotin(IV) derivatives of (5′-GMP)^2−^.

The three possible isomers of R_3_SnL (where L = bidentate ligand) have been reported [17] to have different QS values: QS for isomer (a) 1.7–2.3 mm s^−1^; for (b) 3.0–3.9 mm s^−1^; and for (c) 3.5–4.1 mm s^−1^ (Figure 1). Therefore, on the basis of the QS values, the geometry adopted by all of the triorganotin(IV) derivatives would be similar to that as shown in Figure 1(b). The slightly low value of QS (2.52 mm s^−1^) for triphenyltin(IV) derivatives is in accordance with the reported the fact that QS and IS values decrease when an alkyl group is replaced by a phenyl group. Therefore, polymeric structures involving a bidentate phosphate group in axial position and three organic groups in equatorial position leading to either 2- or 3-dimensional associated lattice have been proposed for triorganotin(IV) derivatives of (5′-GMP)^2−^ as shown in Figure 2. A monomeric structure involving a four coordinate R_3_Sn(IV) moiety bonded individually to (PO_3_)^2−^ and 3′-O has been ruled out on the basis of the presence of only one tin species in ^119^Sn Mössbauer spectra with *ρ* value greater than four.

**Figure 3:**
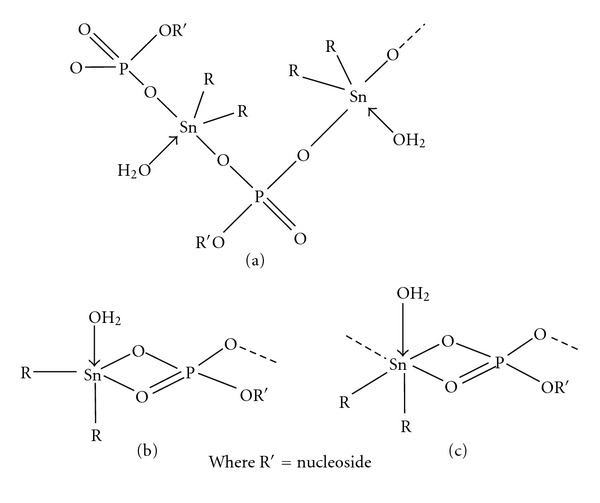
Proposed structures of diorganotin(IV) derivatives of (5′-GMP)^2−^.

A considerable number of possible structures (Figure 3) may be proposed for diorganotin(IV) derivatives of (5′-GMP)^2−^, which correspond to a distorted trigonal-bipyramidal geometry involving one water molecule with either two axial or axial-equatorial disposition of both organic groups and a bidentate phosphate group (Figure 3(a) and Figure 3(b)), and a distorted *cis*-octahedral geometry (Figure 3(c)). The structure as shown in Figure 3(c) may be ruled out on the basis of ^119^Sn NMR chemical shift (discussed later) corresponding to five-coordinated tin (Table 2).

The characteristic resonances in the ^1^H, ^13^C, and ^119^Sn NMR spectral data of the studied di- and triorganotin(IV) derivatives of (5′-GMP)^2−^, recorded in dimethyl-sulfoxide-*d*_6_, are presented in Table 2. The ^1^H NMR spectral data of Na_2_(5′-GMP) are also included in Table 2 for comparison. In di- and triorganotin(IV) derivatives of (5′-GMP)^2−^, all the resonances of (5′-GMP)^2−^ are observed at the expected position as compared to Na_2_(5′-GMP). The H-5′ resonances are considerably shifted indicating the involvement of (PO_3_)^2−^ group in bonding with organotin(IV) moiety. The resonances observed due to the tin-alkyl/phenyl protons in the studied organotin(IV) derivatives of (5′-GMP)^2−^ are observed in the expected regions. The downfield shifts in N(1)-H and NH_2_ resonances may be due to the different extent of hydrogen bonding in the studied derivatives.

The ^13^C NMR spectra of [*n*-Bu_2_Sn(5′-GMP)·H_2_O]_*n*_ and [(Me_3_Sn)_2_(5′-GMP)·H_2_O]_*n*_ could not be recorded because of their extremely low solubility in DMSO-*d*_6_/CDCl_3_/CD_3_OD. The chemical shifts of various magnetically nonequivalent carbons of (5′-GMP)^2−^ have been assigned in the studied derivatives. The C-5′ resonances in organotin(IV) derivatives of (5′-GMP)^2−^ are shifted towards downfield upon complexation as compared with that of ligand, which indicate the involvement of phosphate group (PO_3_)^2−^ in bonding with tin. While all other carbon shifts remains almost unchanged, the ^13^C chemical shifts of alkyl and phenyl groups attached to tin are also observed in the expected regions which are consistent with previously reported values [13, 29]. The characteristic resonances in the ^119^Sn NMR spectra of some of the studied derivatives, recorded in dimethylsulfoxide-*d*_6_, are also presented in Table 2. The satisfactory ^119^Sn NMR spectra of **1, 2, 4,** and** 6** could not be recorded due to their poor solubility even in DMSO-*d*_6_. The ^119^Sn chemical shifts in *iso*-Pr_3_Sn(IV), Ph_2_Sn(IV), and Ph_3_Sn(IV) derivatives of (5′-GMP)^2−^ are observed at *δ*  −256, −225, and −226 ppm, which are characteristic of the five-coordinated organotin(IV) derivatives [13, 17, 21, 22, 32].

The anti-inflammatory activity (% inhibition) and toxicity data of di- and triorganotin(IV) derivatives of (5′-GMP)^2−^ are presented in Table 3. The activity of the studied derivatives is influenced by the nature of the ligand and the organic groups attached to tin. Organotin(IV) derivatives of (5′-GMP)^2−^ show better activity as compared to those of guanosine (~7.51–9.21% inhibition at 40 mg kg^−1^ dose) [22], whereas di- and triorganotin(IV) derivatives of (5′-GMP)^2−^ displayed mild-to-moderate anti-inflammatory activity (~15.64–20.63% inhibition at 40 mg kg^−1^ dose) which is significantly lower than that of phenylbutazone (34.56% inhibition). It has been observed that the activity decreases with the increases in size of the alkyl group, that is, Me_2_Sn(IV) derivative is better than *n*-Bu_2_Sn(IV), and *iso*-Pr_3_Sn(IV) derivative is better than *n*-Bu_3_Sn(IV) derivative. Further, phenyltin(IV) derivatives show better activity as compared to their alkyl analogues. Furthermore, triorganotin(IV) derivatives of guanosine and (5′-GMP)^2−^ show slightly higher activity than the corresponding diorganotin(IV) derivatives. [(Ph_3_Sn)_2_(5′-GMP)·H_2_O]_*n*_ exhibited the highest anti-inflammatory activity (20.63% inhibition) among the studied derivatives. The higher activity of diphenyltin(IV) and triphenyltin(IV) derivatives of (5′-GMP)^2−^ among the studied derivatives may be due to the formation and frequent transportation of Ph_2_Sn(IV)^2+^/Ph_3_Sn(IV)^+^ moiety across the cellular membrane as part of the mechanism for inhibition.

The observed LD_50_ values (Table 3) indicate that di- and triorganotin(IV) derivatives of (5′-GMP)^2−^ are less toxic (LD_50_  >  400 mg kg^−1^) than the corresponding derivatives of guanosine (LD_50_  >  200 mg kg^−1^) [22]. Further, it has been observed that the LD_50_ values of the studied derivatives are comparable (>400 mg kg^−1^) with those of other compounds reported earlier [33] and much higher than those of the diorganotin(IV) derivatives of the simple *α*-amino acids (<50 mg/kg) [34], indicating that the bigger biomolecules lower the toxicities. 

## Supplementary Material

The ^119^Sn Mössbauer spectrum of [(*n*-Bu_3_Sn)_2_(5'-GMP).H_2_O]_n_ (Suppl. Fig. 1) clearly indicates the geometry around tin would be similar to that as shown in Figure 1(b) and corresponds to polymeric structures involving a bidentate phosphate group in axial position and three butyl groups in equatorial position leading to either 2- or 3-dimensional associated lattice as shown in Figure 2 in the text. Further, ^119^Sn Mössbauer spectrum of [Me_2_Sn(5'-GMP).H_2_O]_n_ corresponds to a distorted trigonal-bipyramidal geometry around tin involving one water molecule with either two axial or axial-equatorial disposition of both organic groups and a bidentate phosphate group (as shown in Fig. 3(a) and Fig. 3(b) of Text). ^1^H and ^13^C NMR (Suppl. Fig. 3 and Fig. 4) also clearly indicate all the possible resonances of the different groups, and ^119^Sn NMR spectrum of [(Ph_3_Sn)_2_(5'-GMP).H_2_O]_n_ indicates five-coordinated organotin(IV) derivative (-226 ppm).

## Figures and Tables

**Scheme 2:**
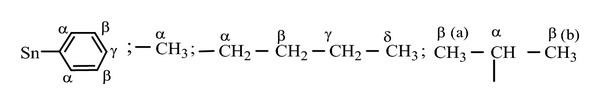


**Table 1:** ^119^Sn Mössbauer data (80 K) of di- and triorganotin(IV) derivatives of (5′-GMP)^2−^.

Complex	(Q.S.)^a^	(I.S.)^a^	*ρ*	*τ*_1_(L)	*τ*_2_(R)
	(mm s^−1^)	(mm s^−1^)	(Q.S./I.S.)		
[Me_2_Sn(5′-GMP)·H_2_O]_*n*_	3.55	1.14	3.11	2.00	2.00
[*n*-Bu_2_Sn(5′-GMP)·H_2_O]_*n*_	3.24	1.15	2.82	2.42	2.27
[Ph_2_Sn(5′-GMP)·H_2_O]_*n*_	1.83	0.61	3.00	2.70	3.18
[(*i*-Pr_3_Sn)_2_(5′-GMP)·H_2_O]_*n*_	3.35	1.40	2.39	1.88	2.00
[(*n*-Bu_3_Sn)_2_(5′-GMP)·H_2_O]_*n*_	3.30	1.40	2.36	1.16	1.27
[(Ph_3_Sn)_2_(5′-GMP)·H_2_O]_*n*_	2.52	0.95	2.65	3.00	3.63

^a^QS: quadrupole splitting; IS: isomeric shift relative to BaSnO_3_ and tin foil (splitting: 2.52 mm s^−1^); *τ*_1_(L): half line-width left doublet component; *τ*_2_(R): half line-width right doublet component (mm s^−1^).

**Table 2:** ^1^H,  ^13^C, and  ^19^Sn NMR spectral data of di- and triorganotin(IV) derivatives of (5′-GMP)^2−^ at 300 MHz in DMSO-d_6_.

Ligand/Complex no.^a^	*δ* (ppm)^b^
Na_2_(5′-GMP) (500 MHz)	N(1)-H: 10.67 (s, 1H); NH_2_: 6.48 (s, 2H); H-8: 7.95 (s, 1H); H-1′: 5.70 (d, 5.0 Hz, 1H); H-2′: 4.40 (s, 1H); 2′-OH: 5.42 (d, 5.5 Hz) and 5.14 (d, 3.0 Hz, 1H); H-3′: 4.09 (d, 4.0 Hz, 1H); 3′-OH: 5.06 (t, 4.5 Hz, 1H); H-4′: 3.87 (d, 3.0 Hz, 1H); H-5′: 3.55−3.51 (m)^d^ and 3.64−3.60 (m, 2H); C-2: 153.6; C-4: 151.3; C-5: 116.6; C-6: 156.7; C-8: 135.6; C-1′: 86.3; C-2′: 73.6; C-3′: 70.3; C-4′: 85.2; C-5′: 61.3

[Me_2_Sn(5′-GMP)·H_2_O]_*n*_	N(1)-H: 10.74 (s, 1H); NH_2_: 6.52 (s, 2H); H-8: 7.95 (s, 1H); H-1′: 5.71 (s, 1H); H-2′: 4.44 (s, 1H); 2′-OH: 5.41 (s, 1H); H-3′: 4.14 (s, 1H); 3′-OH: 5.26 (s, 1H); H-4′: 4.00 (br s, 1H); H-5′: 3.46 (d, 3.0 Hz, 2H); H-*α*: 0.63, 0.99 (s, 6H)^c^; C-2: 153.8; C-4: 151.9; C-5: 116.3; C-6: 157.2; C-8: 135.6; C-1′: 85.9; C-2′: 74.2; C-3′: 71.3; C-4′: 84.2; C-5′: 65.4; C-*α*: 11.8, 13.9

[*n*-Bu_2_Sn(5′-GMP)·H_2_O]_*n*_	N(1)-H: 10.74 (s, 1H); NH_2_: 6.63 (t, 3.5 Hz, 2H); H-8: 7.91 (s, 1H); H-1′: 5.76 (s, 1H); H-2′: 4.16 (s, 1H); 2′-OH: 5.53 (s, 1H); H-3′: 4.03 (s, 1H); 3′-OH: 4.48 (s, 1H); H-4′: 3.93 (br s, 1H); H-5′: 3.52 (s), 3.43 (d, 3.0 Hz, 2H); H-*α* and H-*γ*: 1.34 (m, 8H)^d^; H-*β*: 1.69 (m, 4H); H-*δ*: 0.91 (t, 6H)

[Ph_2_Sn(5′-GMP)·H_2_O]_*n*_	N(1)-H: 10.76 (s, 1H); NH_2_: 6.58 (s, 2H); H-8: 7.92 (s, 1H); H-1′: 5.79 (s, 1H); H-2′: 4.46 (s, 1H); 2′-OH: 5.57 (s, 1H); H-3′: 4.10 (s, 1H); 3′-OH: 5.38 (s, 1H); H-4′: 3.91 (s, 1H)^c^; H-5′: 3.66 (br d, 2H); H-*α*: 7.80 (d, 7.1 Hz, 4H); H-*β*: 7.50 (d, 7.5 Hz, 4H); H-*γ*: 7.32 (br m, 2H); C-2: 153.9; C-4: 151.6; C-5: 116.5; C-6: 156.9; C-8: 135.1; C-1′: 86.0; C-2′: 73.6; C-3′:70.6; C-4′: 83.1; C-5′: 65.9; C-*i*: 140.8; C-*α*: 136.1; C-*β*: 128.5; C-*γ*: 129.2; ^119^Sn: −225.1

[(Me_3_Sn)_2_(5′-GMP)·H_2_O]_*n*_	N(1)-H: 10.67 (s, 1H); NH_2_: 6.49 (s, 2H); H-8: 7.93 (s, 1H); H-1′: 5.70 (s, 1H); H-2′: 4.44 (s, 1H); 2′-OH: 5.37 (s, 1H); H-3′: 4.15 (s, 1H); 3′-OH: 5.22 (s, 1H); H-4′: 3.98 (d, 7.5 Hz, 1H); H-5′: 3.36 (br d, 2H); H-*α*: 0.63, 0.76, 0.98 (s, 18H)

[(*i*-Pr_3_-Sn)_2_(5′-GMP)·H_2_O]_*n*_	N(1)-H: 10.76 (s, 1H); NH_2_: 6.60 (s, 2H); H-8: 7.73 (s, 1H); H-1′: 5.68 (d, 6.0 Hz, 1H); H-2′: 4.36, (4.48) (s, 1H); 2′-OH: 5.55 (s, 1H); H-3′: 4.07 (s, 1H); 3′-OH: 5.41 (s, 1H); H-4′: 3.94 (br s, 1H); H-5′: 3.73 (br s, 2H); H-*α*: 1.08 (m, 6H); H-*β* (a): 1.65 (d, 9.0 Hz, 18H); H-*β* (b): 0.90 (t, 6.0 Hz, 18 H); C-2: 153.8; C-4: 151.5; C-5: 116.5; C-6: 156.9; C-8: 134.7; C-1′: 85.8; C-2′: 73.7; C-3′: 70.8; C-4′: 83.6; C-5′: 64.4; C-*α*: 24.2; C-*β* (a): 19.0, (19.2); C-*β* (b): 18.8, (18.5); ^119^Sn: −255.7

[(*n*-Bu_3_Sn)_2_(5′-GMP)·H_2_O]_*n*_	N(1)-H: 10.67 (s, 1H); NH_2_: 6.51 (s, 2H); H-8: 7.62 (s, 1H); H-1′: 5.69 (s, 1H); H-2′: 4.26 (s, 1H); 2′-OH: 5.50 (s, 1H); H-3′: 3.96 (d, 18 Hz, 1H); 3′-OH: 5.11 (br s, 1H); H-4′: 3.71 (br s, 1H); H-5′: 3.40 (s, 2H); H-*α*: 1.05 (s, 12H); H-*β*: 1.57 (s, 12H); H-*γ*: 1.26 (d, 6.0 Hz, 12H); H-*δ*: 0.82 (s, 18H); C-2: 153.8; C-4: 151.6; C-5: 116.5; C-6: 156.9; C-8: 134.2; C-1′: 85.7; C-2′: 73.9; C-3′: 70.9; C-4′: 83.4; C-5′: 64.7; C-*α*: 19.9; C-*β*: 27.8; C-*γ*: 26.8 [83.0 Hz]^b^; C-*δ*: 13.7

[(Ph_3_Sn)_2_(5′-GMP)·H_2_O]_*n*_	N(1)-H: 10.66 (s, 1H); NH_2_: 6.51 (s, 2H); H-8: 7.75 (s, 1H); H-1′: 5.69 (s, 1H); H-2′: 4.41 (s, 1H); 2′-OH: 5.42 (s, 1H); H-3′: 4.10 (s, 1H); 3′-OH: 5.19 (s, 1H); H-4′: 3.96 (s, 1H); H-5′: 3.41, (3.17), (s, 2H); H-*α* + H-*β* + H-*γ*: 7.00–7.32 (br m, 30H)^d^; C-2: 153.7; C-4: 151.9; C-5: 116.5; C-6: 156.9; C-8: 135.1; C-1′: (85.1); C-2′: (73.6); C-3′: (70.4); C-4′: (83.1); C-5′: (65.0); C-*i*: 142.8, 142.1; C-*α*: 136.2, 136.1; C-*β*: 128.0, (127.7); C-*γ*: 128.3, (128.8); ^119^Sn: −225.8

^a^According to Experimental section; ^b^homonuclear proton-proton coupling multiplet abbreviations given in parentheses: s: singlet; d: doublet; t: triplet; br: broad; m: multiplet; ^c^fused singlet; ^d^overlapping multiplets; weak signals in parantheses; resonances H-*β* (a) and H-*β* (b) may be interchanged(see Scheme 2).

**Table 3:** Anti-inflammatory activity and toxicity data of di- and triorganotin(IV) derivatives of (5′-GMP)^2−^.

Complex/standard drug	Anti-inflammatory activity^a^		Toxicity LD_50_
	Dose (mg/kg p.o.)	% inhibition	mg/kg p.o.
[Me_2_Sn(5′-GMP)·H_2_O]_*n*_	40	18.12	>400
[*n*-Bu_2_Sn(5′-GMP)·H_2_O]_*n*_	40	16.32	>400
[Ph_2_Sn(5′-GMP)·H_2_O]_*n*_	40	19.22	>400
[(*i*-Pr_3_Sn)_2_(5′-GMP)·H_2_O]_*n*_	40	15.64	>400
[(*n*-Bu_3_Sn)_2_(5′-GMP)·H_2_O]_*n*_	40	17.34	>400
[(Ph_3_Sn)_2_(5′-GMP)·H_2_O]_*n*_	40	20.63	>400
Phenyl butazone	40	34.56	>2000

^a^% inhibition in paw edema = [(DC − DT)/DC] × 100, where DT and DC are the mean volumes of paw edema in drug-treated and control groups. Standard deviation (*σ*) in DC is <0.09 and in DT <0.15.

Standard error in mean [(SEM) = (*σ*/(*N*)^1/2^)] in DC is <0.040 and in DT <0.067.
