# Supplementary figures and images for: Interaction of 5′-Guanosine Monophosphate with Organotin(IV) Moieties: Synthesis, Structural Characterization, and Anti-Inflammatory Activity
Source: ISRN Org Chem. 2012 Sep 30;2012:873035. doi: 10.5402/2012/873035 (PMC3767334; doi:10.5402/2012/873035)

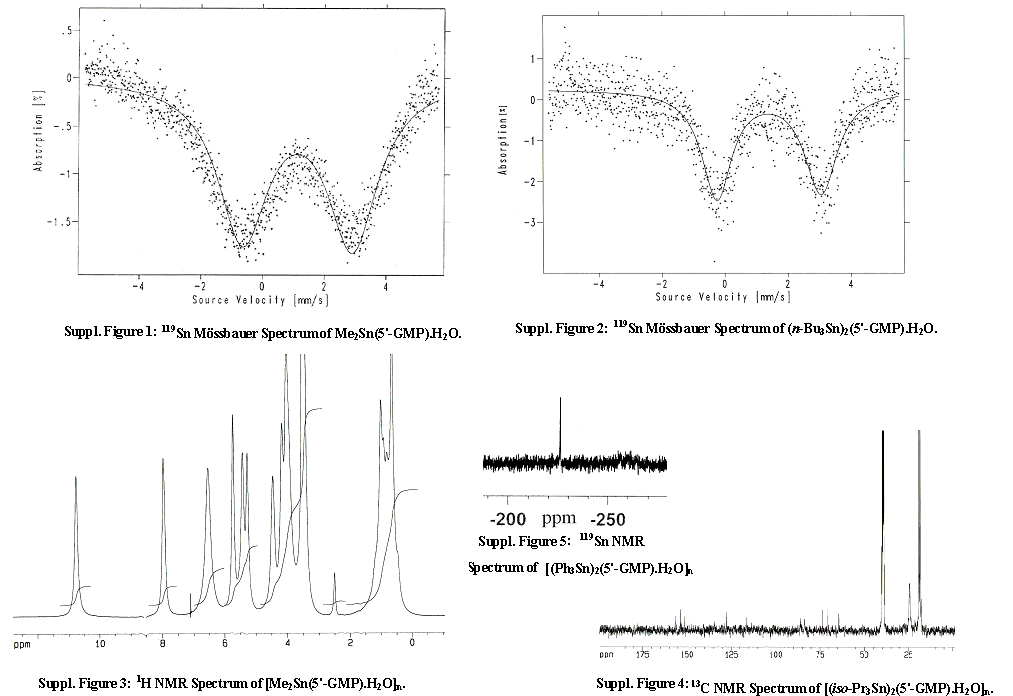

Supplement: Supplementary file 1 — The 119Sn Mössbauer spectrum of [(n-Bu3Sn)2(5'-GMP).H2O]n (Suppl. Fig. 1) clearly indicates the geometry around tin would be similar to that as shown in Figure 1(b) and corresponds to polymeric structures involving a bidentate phosphate group in axial position and three butyl groups in equatorial position leading to either 2- or 3-dimensional associated lattice as shown in Figure 2 in the text. Further, 119Sn Mössbauer spectrum of [Me2Sn(5'-GMP).H2O]n corresponds to a distorted trigonal-bipyramidal geometry around tin involving one water molecule with either two axial or axial-equatorial disposition of both organic groups and a bidentate phosphate group (as shown in Fig. 3(a) and Fig. 3(b) of Text). 1H and 13C NMR (Suppl. Fig. 3 and Fig. 4) also clearly indicate all the possible resonances of the different groups, and 119Sn NMR spectrum of [(Ph3Sn)2(5'-GMP).H2O]n indicates five-coordinated organotin(IV) derivative (-226 ppm). [file 873035.f1.tif]
